# The Ketimide Ligand is Not Just an Inert Spectator: Heteroallene Insertion Reactivity of an Actinide–Ketimide Linkage in a Thorium Carbene Amide Ketimide Complex**

**DOI:** 10.1002/anie.201404898

**Published:** 2014-07-07

**Authors:** Erli Lu, William Lewis, Alexander J Blake, Stephen T Liddle

**Affiliations:** School of Chemistry, University of Nottingham, University ParkNottingham, NG7 2RD (UK)

**Keywords:** amides, carbenes, ketimides, N ligands, thorium

## Abstract

The ketimide anion R_2_C—N^−^ is an important class of chemically robust ligand that binds strongly to metal ions and is considered ideal for supporting reactive metal fragments due to its inert spectator nature; this contrasts with R_2_N^−^ amides that exhibit a wide range of reactivities. Here, we report the synthesis and characterization of a rare example of an actinide ketimide complex [Th(BIPM^TMS^){N(SiMe_3_)_2_}(N—CPh_2_)] [**2**, BIPM^TMS^=C(PPh_2_NSiMe_3_)_2_]. Complex **2** contains Th—C_carbene_, Th—N_amide_ and Th—N_ketimide_ linkages, thereby presenting the opportunity to probe the preferential reactivity of these linkages. Importantly, reactivity studies of **2** with unsaturated substrates shows that insertion reactions occur preferentially at the Th—N_ketimide_ bond rather than at the Th—C_carbene_ or Th—N_amide_ bonds. This overturns the established view that metal-ketimide linkages are purely inert spectators.

Amide (R_2_N^−^) and ketimide (R_2_C—N^−^) (R=alkyl, aryl, or silyl groups) monoanions are two important classes of monodentate nitrogen-donor ligands in coordination and organometallic chemistry. The negative charge of both these types of ligand is N-centered and can form a covalent M—N bond in metal complex derivatives. However, there is a crucial difference between amides and ketimides: in the former the nitrogen hybridization is sp^2^ or sp^3^ and it bears two N—C/Si singly bonded groups, whereas in the latter the nitrogen hybridization is sp or sp^2^ and it is bonded to only one carbon atom with a N—C double bond. These differences in structure and bonding lead to a significantly different reactivity of these two types of M—N bond. The M—N_amide_ bond is reactive, and readily engages in protonolysis and insertion of unsaturated organic substrates; this has been extensively studied for decades and these reactions play a vital role in very important catalytic processes such as hydroamination, hydroalkoxylation, and ring-opening polymerization of lactones.[1] In sharp contrast, the M—N_ketimide_ bond is chemically inert, and resistant to insertion and electrophilic attack.[2] In fact, the chemically inert nature of M—N_ketimide_ bonds renders ketimides the ligand of choice when spectator ligands are required to stabilize highly reactive species in a broad range of applications including strongly oxidizing high-valent uranium and Group 7–9 complexes,[3] diuranium inverted-sandwich arene complexes,[4] and olefin polymerization catalysts.[5] To the best of our knowledge, the only reported reactivity of any metal–ketimide, in the absence of acidic hydrogens, involves β-R-group eliminations and free-radical redox C—C bond homolysis degradation reactions of the ketimide rather than M—N_ketimide_ insertion chemistry.[6]

Nonaqueous actinide chemistry has received burgeoning interest in the past decade.[7] Although actinide amides are less well-developed than their transition metal counterparts, they have been known for decades and their reactivity is extensively investigated.[8]–[10] In contrast, actinide ketimides were unknown until 2002. After the first example of a uranium ketimide,[11] a relatively small number of actinide ketimides have been synthesized and characterized.[12] Studies of the An—N_ketimide_ (An=U, Th) bond have shed light on the important question of the amount of 5f orbital participation in bonding.[13] However, from a reactivity perspective, and as compared to their transition metal counterparts, the An—N_ketimide_ bond is generally considered to be chemically inert,[11] and considerably stronger than analogous An—N_amide_ bonds. Indeed, no insertion reactivity of the An—N_ketimide_ spectator ligand linkage with a wide range of substrates has ever been observed,[11], [12] despite the fact that the An—N_ketimide_ linkage is usually the least sterically hindered linkage in An-complexes. Furthermore, from a general perspective, a direct comparison of bonding character and reactivity of M—N_amide_/M—N_ketimide_ linkages in one molecule is desirable but still absent. Such studies may provide information on potential catalytic mechanisms and/or deactivation pathways of such complexes, and open new horizons for M—N linkage reactivity.

As part of our investigations of An-ligand multiple bond chemistry,[14] we describe here the synthesis of a thorium carbene amide ketimide that features Th—C_carbene_, Th—N_amide_, and Th—N_ketimide_ bonds in one molecule. Preliminary reactivity studies unexpectedly revealed that insertion reactions occur at the traditionally inert M—N_ketimide_ site, rather than at the M—C_carbene_[15] or M—N_amide_ bonds. This observation overturns the view that ketimides are purely inert spectator ligands.

To begin with, [ThCl_4_(DME)_2_][16] was treated with one equivalent of Li_2_BIPM^TMS^ [BIPM^TMS^=C(PPh_2_NSiMe_3_)_2_] to form the thorium dichloride intermediate [Th(BIPM^TMS^)(Cl)_2_].[17] This intermediate was not isolated and treated with one equivalent of KN(SiMe_3_)_2_ in situ. After work-up and recrystallization, the thorium carbene amide chloride [Th(BIPM^TMS^){N(SiMe_3_)_2_}(μ-Cl)]_2_ (**1**) was obtained in 85 % yield as a pale yellow solid (Scheme 1). Although a number of covalent uranium carbenes have been reported in recent years,[14], [18] thorium analogues remain exceptionally rare.[19] Treatment of **1** with two equivalents of [Li(N—CPh_2_)] in benzene at room temperature results in a color change from pale yellow to intense orange. Because of the 6d^0^5f^0^ metal ion configuration, Th^IV^ complexes have usually been reported as essentially colorless. After the work-up, the thorium carbene amide ketimide [Th(BIPM^TMS^){N(SiMe_3_)_2_}(N—CPh_2_)] (**2**) was isolated as orange crystals in 91 % yield (Scheme 1).

**Scheme 1:**
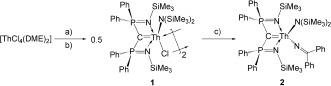
Synthesis of 1 and 2. Reagents and conditions: a) Li_2_BIPM^TMS^, THF, −78 °C, −2 LiCl; b) KN(SiMe_3_)_2_, C_6_H_6_, RT, −KCl; c) LiN—CPh_2_, C_6_H_6_, RT, −LiCl.

The characterization data for **1** and **2** are consistent with their formulations.[17] The vivid orange color of **2** (both in the solid-state and in solution) is noteworthy for a 6d^0^5f^0^ metal complex. The electronic absorption spectra of **2** exhibits a broad, intense electronic absorption band from the UV to visible wavelength range, and a strong absorption between 450 and 550 nm. Since **1** is pale yellow and the 6d^0^5f^0^ electronic configuration of Th^IV^ precludes metal-localized f–f, d–f, and d–d transitions, and on the basis of definitive prior work,[12c] we conclude this transition is due to a spin-allowed but orbital-forbidden p⊥(N)→π* (N—C) and ligand-to-metal charge transfer (LMCT).

The solid-state structures of **1** and **2** were confirmed by X-ray crystallography (**1**, Figure S1; **2**, Figure 1). The salient structural feature of **2** is the two types of Th—N linkage; the Th—N_ketimide_ distance is significantly shorter than the Th—N_amide_ bond (Th–N4 2.265(6) Å versus Th–N3 2.350(7) Å). The ketimide N—C bond length is 1.279(10) Å, and Th-N-C bond angle is 171.3(6)°. These parameters suggest that the Th—N_ketimide_ interaction may be stronger than the Th—N_amide_ interaction,[11] and may feature some multiple bond character. The Th—C_carbene_ bond lengths in **1** and **2** are 2.410(8) Å and 2.474(8) Å, respectively, which is similar to other thorium BIPM carbene complexes (2.43–2.50 Å).[19] Although the M—C bond in An and rare-earth BIPM carbene complexes is polarized,[14], [18] a modest multiple bond character of the Th—C_carbene_ bonds in **1** and **2** is suggested by comparison to the thorium alkyl complex [Th(CH_2_CMe_3_)_5_][Li(THF)_4_],[20] in which the Th—C single bond (2.50–2.56 Å) is longer than the Th—C bonds in **1** or **2**.

**Figure 1:**
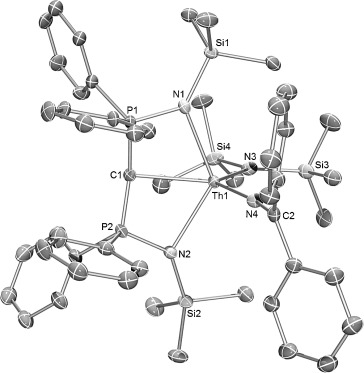
Molecular structure of [Th(BIPM^TMS^){N(SiMe_3_)_2_}(N—CPh_2_)] (2). Displacement ellipsoids set at 40 % probability. Hydrogen atoms and minor disorder components are omitted for clarity. Selected bond lengths [Å] and angles [°]: Th–C1 2.474(8), Th–N3 2.350(7), Th–N4 2.265(6), N4–C2 1.279(10), Th–N1 2.431(7), Th–N2 2.429(6); Th-N4-C2 171.3(6).

The presence of Th—C_carbene_, Th—N_amide_, and Th—N_ketimide_ bonds in **2** offers the opportunity to probe their competitive reactivity toward unsaturated organic molecules. For the M—C bond in An and rare-earth metal carbene complexes with BIPM ligands, the cycloaddition and Wittig-type group transfer reaction towards unsaturated organic substrates containing C—E (E=O, N) bonds has been well-documented, even in complexes that can be considered as sterically saturated.[14], [15], [18] On the other hand, M—N_amide_ bonds (M=d- or f-block metal) are also known to undergo a wide range of reactions with unsaturated substrates. Moreover, irrespective of the predicted reactivity of the Th—C_carbene_ and Th—N_amide_ linkages, the Th—N_ketimide_ bond would be anticipated to be inert. However, we find that when **2** is reacted with one equivalent of aldehyde or isocyanate, insertion reactions occur at the Th—N_ketimide_ linkage (Scheme 2).

**Scheme 2:**
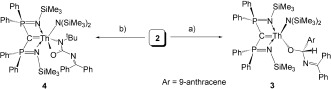
Reactions of 2 with 9-anthracene carboxaldehyde or *t*BuNCO to give 3 and 4. Reagents and conditions: a) ArCHO, toluene, RT; b) *t*BuNCO, toluene, RT.

When **2** was treated with one equivalent of 9-anthracene carboxaldehyde in C_6_D_6_ at room temperature, the orange color of **2** faded into pale yellow within 12 h. ^1^H and ^31^P NMR spectroscopic monitoring of the reaction showed that **2** was completely converted into the new complex [Th(BIPM^TMS^){N(SiMe_3_)_2_}{OC(H)(NCPh_2_)(C_14_H_9_)}] (**3**) within 12 h. The reaction was scaled up with toluene as solvent, providing **3** as yellow crystals in 61 % yield (Scheme 2);[17] this moderate crystalline yield is due to the high solubility of **3** in toluene and not the production of other side-products in the reaction. Unlike **2**, **3** is pale-colored and has no significant absorptions in its electronic absorption spectrum in the UV/Vis range, which is consistent with the loss of the Th—N_ketimide_ bond. Single-crystals suitable for X-ray crystallographic study were obtained from a toluene/hexane mixture, and X-ray analysis confirmed that **3** is a thorium carbene amide alkyloxide derivative (Figure 2) arising from the selective insertion of the C—O bond of 9-anthracene carboxaldehyde into the Th—N_ketimide_ bond.

**Figure 2:**
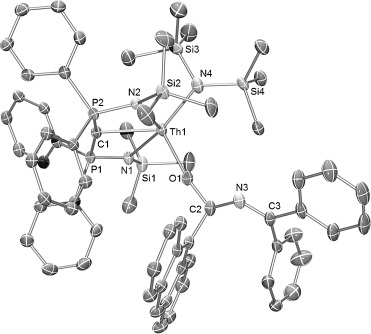
Molecular structure of [Th(BIPM^TMS^){N(SiMe_3_)_2_}{OC(H)(NCPh_2_)(C_14_H_9_)}] (3). Displacement ellipsoids set at 40 % probability. Hydrogen atoms, minor disorder components, and toluene solvent molecule in lattice are omitted for clarity. Selected bond lengths [Å]: Th–C1 2.453(4), Th–O 2.166(3), Th–N1 2.453(4), Th–N2 2.460(4), Th–N4 2.359(4), O–C2 1.392(6), C2–N3 1.471(6), N3–C3 1.290(7).

Isocyanate is an important heteroallene with versatile reactivity in organic and polymer synthesis and insertions of isocyanates into M—N_amide_ bonds in the d-block are widely reported.[21] We have previously shown that the M—C bonds (M=lanthanide or uranium) in BIPM derivatives readily undergo [2+2] cycloaddition reactions with the C—O bond.[14l, 15] When **2** was treated with one equivalent of *tert*-butyl isocyanate (*t*BuN—C—O) in toluene at room temperature, pale-yellow crystals of [Th(BIPM^TMS^){N(SiMe_3_)_2_}{OC(N*t*Bu)NCPh_2_}] (**4**) were obtained in 49 % yield (Scheme 2). The moderate crystalline yield is due to the high solubility of **4** in toluene, and **4** was confirmed to be the single product by an NMR-scale reaction with >95 % conversion. The structure of complex **4** was confirmed by X-ray crystallography as a thorium carbene amide ureate (Figure 3). In this instance, the Th—N_ketimide_ bond was again shown to be active in insertion chemistry. The ureate ligand, which is formed by the selective insertion of C—O into the Th—N_ketimide_ bond, is coordinated to the thorium center in a κ^2^-O, N manner.

**Figure 3:**
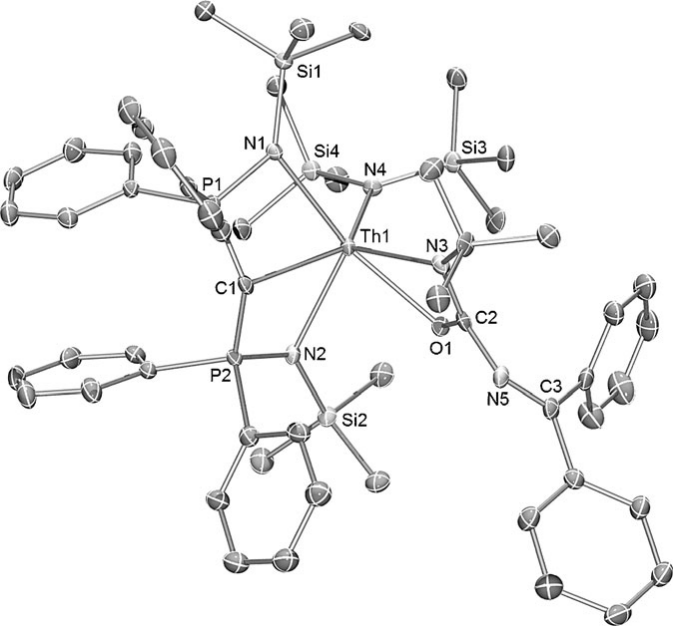
Molecular structure of [Th(BIPM^TMS^){N(SiMe_3_)_2_}{OC(N*t*Bu)NCPh_2_}] (4). Displacement ellipsoids set at 40 % probability. Hydrogen atoms are omitted for clarity. Selected bond lengths [Å]: Th–C1 2.463(5), Th–N1 2.531(4), Th–N2 2.483(5), Th–N3 2.491(5), Th–N4 2.344(4), Th–O 2.391(4), O–C2 1.304(7), C2–N3 1.316(8), C2–N5 1.392(8), N5–C3 1.288(8).

To address the issue of whether **3** or **4** can react further we treated them with one more equivalent of 9-anthracene carboxaldehyde (for **3**) or *t*BuNCO (for **4**) in C_6_D_6_ solvent. In case of **3**, heating at 60 °C leads to the slow formation of the alkene Wittig-product ArC(H)—C(PPh_2_NSiMe_3_)_2_.14b In case of **4**, heating to 60 °C resulted in an intractable mixture and decomposition. These results indicate that the Th—N_amide_ and Th—C_carbene_ bonds in **2** are more resistant towards chemical transformations than the Th—N_ketimide_, which is the opposite of what would be expected.

To conclude, a thorium carbene amide ketimide bearing Th—C_carbene_, Th—N_amide_, and Th—N_ketimide_ linkages has been synthesized and fully characterized. A comparative study of these linkages has shown that, in contrast to the established view, the Th—N_ketimide_ bond is not an inert spectator and can in fact engage in insertion reactivity. These results open a new horizon of reactivity for M—N_ketimide_ linkages, and suggest that in a wider context the role of the ketimide ligand in coordination and organometallic chemistry as a reactive functional group, instead of just being an inert supporting ligand, deserves consideration. Further studies toward using this methodology to incorporate the ketimide group into organic molecules are underway.
