# Supplementary material for: The Ketimide Ligand is Not Just an Inert Spectator: Heteroallene Insertion Reactivity of an Actinide–Ketimide Linkage in a Thorium Carbene Amide Ketimide Complex
Source: Angew Chem Int Ed Engl. 2014 Jul 7;53(35):9356–9. doi: 10.1002/anie.201404898 (PMC4464536; doi:10.1002/anie.201404898)
Supplement: Supplementary file 1 [file anie0053-9356-sd1.pdf]

Angewandte Chemie
Eine Zeitschrift der Gesellschaft Deutscher Chemiker

Supporting Information

© Wiley-VCH 2014

69451 Weinheim, Germany

The Ketimide Ligand is Not Just an Inert Spectator: Heteroallene Insertion Reactivity of an Actinide–Ketimide Linkage in a Thorium Carbene Amide Ketimide Complex**

*Erli Lu, William Lewis, Alexander J. Blake, and Stephen T. Liddle**

anie_201404898_sm_miscellaneous_information.pdf

Experimental

General

All manipulations were carried out using Schlenk techniques, or an MBraun UniLab glovebox, under an atmosphere of dry nitrogen. Solvents were dried by passage through activated alumina towers and degassed before use. All solvents were stored over potassium mirrors except for ethers which were stored over activated 4 Å sieves. Deuterated solvent was distilled from potassium, degassed by three freeze-pump-thaw cycles and stored under nitrogen. $[ThCl_4(DME)_2]$[1] and $[LiN{=}CPh_2]$[2] were prepared as described previously. 9-anthracene carboxaldehyde (99%) was purchased from Sigma-Aldrich and dried under dynamic vacuum for one day prior to use. *tert*-Butylisocyanate (99%) was purchased from Sigma-Aldrich, dried over activated 4 Å molecular sieve, distilled under vacuum, degassed by freeze-thaw-vacuum, and stored in the glovebox under −35 °C.

^{1}H, ^{13}C, ^{29}Si, and ^{31}P, NMR spectra were recorded on a Bruker 400 spectrometer operating at 400.2, 100.6, 79.5, and 162.0 MHz respectively; chemical shifts are quoted in ppm and are relative to Me_4Si (^{1}H, ^{13}C, ^{29}Si) or external 85% H_3PO_4 (^{31}P). FTIR spectra were recorded on a Bruker Tensor 27 spectrometer. UV/Vis/NIR spectra were recorded on a Perkin Elmer Lambda 750 spectrometer. Data were collected in 1mm path length cuvettes loaded in an MBraun UniLab glovebox and were run versus the appropriate toluene reference solvent. Elemental microanalyses were carried out by Tong Liu at the University of Nottingham.

Preparation of $[Th(BIPM^{TMS})\{N(SiMe_3)_2\}(\mu\text{-}Cl)]_2$ (1)

A solution of $[Li_2(BIPM^{TMS})]$ (2.28 g, 4.0 mmol) in THF (10 ml) was added to a solution of $[ThCl_4(DME)_2]$ (2.20 g, 4.0 mmol) in THF (10 ml) at −78 °C. The pale yellow mixture was stirred at −78 °C for 30 minutes, then was allowed to warm to room temperature with stirring for 2 h. Volatiles were removed *in vacuo* and the resulted pale yellow viscous oil was dissolved in 15 ml of

benzene, then a solution of [$KN(SiMe_3)_2$] (0.80 g, 4.0 mmol) in benzene (15 ml) was added at room temperature. After stirring at room temperature for 12 h, the mixture was filtered, and all volatiles were removed *in vacuo*. The resulting solid was washed with hexane (3 × 5 ml) to afford a pale yellow solid. Yield: 3.30 g, 85%. Recrystallization from a toluene/hexane mixture at room temperature afforded pale yellow crystals of **1**. Anal. Calcd for $C_{74}H_{112}Cl_2N_6P_4Si_8Th_2 \cdot 0.5(C_7H_8)$: C 46.19; H, 5.80; N, 4.17%. Found: C, 46.32; H, 6.07; N, 3.96%. ^{1}H NMR(C_6D_6, 400 MHz, 298 K): δ (ppm) = 0.28 (s, 18 H, Si(C*H₃*)₃), 0.49 (s, 18 H, Si(C*H₃*)₃), 2.11 (s, 1.5 H, C*H₃* of toluene), 6.78 - 6.83 (m, 4 H, Ar*H*), 6.86 - 6.90 (m, 2 H, Ar*H*), 7.00 - 7.03 (m, 1.5 H, Ar*H* of toluene), 7.11 - 7.20 (m, 7.5 H, Ar*H* and Ar*H* of toluene), 7.41 - 7.47 (m, 4 H, Ar*H*), 7.87 - 7.93 (m, 4 H, Ar*H*). ^{13}C NMR (C_6D_6, 100.6 MHz, 298 K): δ (ppm) = 3.01 (s, Si(CH₃)₃), 4.60 (s, Si(CH₃)₃), 21.39 (s, CH₃ of toluene), 80.95 (t, J_{PC} = 153.6 Hz, Th=CP₂), 125.64, 127.86, 127.90, 127.92, 128.51, 128.53, 129.28, 129.84, 130.76 (Ar*C* and Ar*C* of toluene), 131.18 (t, $^3J_{PC}$ = 5.9 Hz, C_{meta} of P–Ph), 132.03 (t, $^3J_{PC}$ = 5.9 Hz, C_{meta} of P–Ph), 135.85 (d, J_{PC} = 44.7 Hz, C_{ipso} of P–Ph), 136.53 (s, Ar*C*), 138.48 (d, J_{PC} = 50.4 Hz, C_{ipso} of P–Ph), 139.24 (s, Ar*C*). ^{31}P NMR (C_6D_6, 162 MHz, 298 K): δ (ppm) = 8.61 (s). ^{29}Si{^{1}H} NMR (C_6D_6, 79.5 MHz, 298 K): δ (ppm) = −6.18 (d, $^2J_{PSi}$ = 3.50 Hz, P=N(*Si*Me₃)), −6.16 (s, N(*Si*Me₃)₂), −6.14 (d, $^2J_{PSi}$ = 3.18 Hz, P=N(*Si*Me₃)). FTIR v/cm^{-1} (Nujol): 1377 (s), 1323 (m), 1106 (m), 933 (s), 802 (m), 722 (s), 694 (w), 605 (w), 544 (w), 525 (w), 496 (w).

Preparation of [Th(BIPM^{TMS}){N(SiMe$_3$)$_2$}(NCPh$_2$)] (2)

A solution of **1** (2.94 g, 1.49 mmol) in benzene (10 ml) was added to a suspension of [$LiNCPh_2$] (0.56 g, 2.98 mmol) in benzene (10 ml) at room temperature. After stirring at room temperature for 12 h, all volatiles were removed *in vacuo*, and the resulted orange viscous oil was extracted by toluene (3 × 5 ml). The extraction was concentrated to approximately 0.5 ml and layered with 3 ml of hexane to afford **2** as orange crystals. Yield: 3.05 g, 91%. Anal. Calcd for $C_{50}H_{66}N_4P_2Si_4Th$: C, 53.17; H, 5.89; N, 4.96%. Found: C, 52.82; H, 6.01; N, 4.52%. ^{1}H NMR (C_6D_6, 300 MHz, 298 K): δ (ppm) = 0.22 (s, 18 H, Si(C*H₃*)₃), 0.51 (s, 18 H, Si(C*H₃*)₃), 6.76 (t, 4 H, $^3J_{HH}$ = 7.5 Hz, *p*-Ar*H*),

6.87 (t, 2 H, $^3J_{HH}$ = 7.2 Hz, *p*-Ar*H*), 7.16 - 7.27 (m, 16 H, Ar*H*), 7.83 - 7.92 (m, 8 H, Ar*H*). ^{13}C NMR (C_6D_6, 100.6 MHz, 298 K): δ (ppm) = 3.51 (s, Si(CH$_3$)$_3$), 4.20 (s, Si(CH$_3$)$_3$), 77.95 (t, J_{PC} = 168.2 Hz, Th=CP$_2$), 127.58, 127.64, 127.85, 127.91, 128.08, 129.02, 129.09, 130.33 (Ar*C*), 130.95 (t, $^3J_{PC}$ = 5.9 Hz, C_{meta} of P–Ph), 131.93 (t, $^3J_{PC}$ = 6.0 Hz, C_{meta} of P–Ph), 137.91 (s, Ar*C*), 138.59 (d, J_{PC} = 45.5 Hz, C_{ipso} of P–Ph), 139.29 (s, Ar*C*), 140.05 (d, J_{PC} = 48.1 Hz, C_{ipso} of P–Ph), 144.93 (s, Ar*C*), 173.84 (s, N*C*Ph$_2$). ^{31}P NMR (C_6D_6, 121.5 MHz, 298 K): δ (ppm) = 5.76 (s). ^{29}Si{^{1}H} NMR (C_6D_6, 79.5 MHz, 298 K): δ (ppm) = −10.52 (s, N(*Si*Me$_3$)$_2$), −7.66 (t, $^2J_{PSi}$ = 4.14 Hz, P=N(*Si*Me$_3$)). UV-Vis (ε, M^{-1} cm^{-1}, toluene): 400 (231), 480 (260). FTIR ν/cm^{-1} (Nujol): 1613 (s), 1592 (m), 1573 (m), 1100 (s, br), 956 (m), 800 (m), 721 (w), 670 (w), 604 (m), 551 (m), 514 (w), 486 (w), 451 (w).

Preparation of [Th(BIPM^{TMS}){N(SiMe$_3$)$_2$}{OC(H)(NCPh$_2$)(C$_{14}$H$_9$)}] (3)

A solution of 9-anthracene carboxaldehyde (0.2 g, 1.0 mmol) in toluene (2.5 ml) was added to a solution of **2** (1.11 g, 1.0 mmol) in toluene (2.5 ml) at room temperature. After stirring at room temperature for 12 h, all volatiles were removed *in vacuo*. The product was recrystallized from a toluene/hexane mixture to yield **3** as yellow crystals. Yield: 0.80 g, 61%. Anal. Calcd for $C_{65}H_{76}N_4OP_2Si_4Th \cdot 0.5\ (C_7H_8)$: C, 59.54; H, 5.84; N, 4.05%. Found: C, 59.36; H, 5.96; N, 4.01%. ^{1}H NMR (C_6D_6, 400 MHz, 298 K): δ (ppm) = 0.09 (s, 9 H, P=N(Si(C*H*$_3$)$_3$)), 0.36 (s, 9 H, P=N(Si(C*H*$_3$)$_3$)), 0.49 (s, 18 H, N(Si(C*H*$_3$)$_3$)$_2$), 2.11 (s, 1.5 H, C*H*$_3$ of toluene), 6.43 - 6.49 (m, 4H, O-C(*H*)N and Ar*H*), 6.61 (t, 2 H, $^3J_{HH}$ = 7.6 Hz, Ar*H*), 6.76 (t, 2 H, $^3J_{HH}$ = 7.6 Hz, Ar*H*), 6.84 - 6.92 (m, 3 H, Ar*H*), 7.00 - 7.07 (m, 3 H, Ar*H*), 7.10 - 7.22 (m, 10.5 H, Ar*H* and Ar*H* of toluene), 7.28 - 7.36 (m, 4 H, Ar*H*), 7.51 - 7.56 (m, 1 H, Ar*H*), 7.75 - 7.90 (m, 8 H, Ar*H*), 8.00 - 8.02 (m, 2 H, Ar*H*), 8.10 (s, 1 H, Ar*H*), 8.22 (s, 1 H, Ar*H*), 10.31 (s, br, 1 H, 9-*H* of anthracene). ^{13}C NMR (C_6D_6, 100.6 MHz, 298 K): δ (ppm) = 3.20 (s, P=N(Si(CH$_3$)$_3$)), 3.41 (s, P=N(Si(CH$_3$)$_3$)), 5.05 (s, N(Si(CH$_3$)$_3$)$_2$), 21.39 (s, CH$_3$ of toluene), 67.25 (t, J_{PC} = 172.4 Hz, Th=CP$_2$), 93.52 (s, O-*C*(H)N), 124.18, 124.41, 125.34, 125.43, 125.64, 127.29, 127.88, 128.45, 128.51, 128.53, 128.95, 129.01, 129.10, 129.27, 129.34, 130.21, 130.45 (Ar*C*), 130.85 (m, Ar*C*), 131.24, 131.61 (Ar*C*), 132.07 (m, Ar*C*), 132.99,

137.39, 138.48, 140.10 (Ar*C*), 164.47 (s, N*C*Ph$_2$). ^{31}P NMR (C_6D_6, 162 MHz, 298 K): δ (ppm) = 5.01 (s). ^{29}Si{^{1}H} NMR (C_6D_6, 79.5 MHz, 298 K): δ (ppm) = −9.96 (m, N(*Si*Me$_3$)$_2$), −7.69 (m, P=N(*Si*Me$_3$)). FTIR v/cm^{-1} (Nujol): 1377 (m), 1050 (m, br), 951 (m), 873 (w), 693 (w), 603 (m), 593 (w), 523 (w).

Preparation of [Th(BIPM^{TMS}){N(SiMe$_3$)$_2$}{OC(N^{t}Bu)NCPh$_2$}] (4)

A solution of ^{t}BuNCO (0.12 g, 1.22 mmol) in toluene (2.5 ml) was added to a solution of **2** (1.37 g, 1.22 mmol) in toluene (2.5 ml) at room temperature. After stirring at room temperature for 24 h, all volatiles were removed *in vacuo*. The product was recrystallized from hexane to yield **4** as pale yellow crystalline solid. Yield: 0.73 g, 49%. Anal. Calcd for $C_{55}H_{75}N_5OP_2Si_4Th$: C, 53.77; H, 6.15; N, 5.70%. Found: C, 53.14; H, 6.10; N, 5.20%. ^{1}H NMR (C_6D_6, 400 MHz, 298 K): δ (ppm) = 0.24 (s, 18 H, N(Si(C*H*$_3$)$_3$)$_2$ or P=N(Si(C*H*$_3$)$_3$)), 0.48 (s, 18 H, N(Si(C*H*$_3$)$_3$)$_2$ or P=N(Si(C*H*$_3$)$_3$)), 1.47 (s, 9 H, NC(C*H*$_3$)$_3$)), 6.84 - 6.88 (m, 4 H, Ar*H*), 6.96 - 7.03 (m, 4 H, Ar*H*), 7.10 - 7.13 (m, 6 H, Ar*H*), 7.16 - 7.25 (m, 4 H, Ar*H*), 7.35 - 7.41 (m, 4 H, Ar*H*), 7.58 - 7.61 (m, 4 H, Ar*H*), 7.91 - 7.97 (m, 4 H, Ar*H*). ^{13}C NMR (C_6D_6, 100.6 MHz, 298 K): δ (ppm) = 4.76 (s, Si(*C*H$_3$)$_3$), 32.16 (s, C(*C*H$_3$)$_3$), 52.71 (s, *C*(CH$_3$)$_3$), 83.05 (t, J_{PC} = 160.1 Hz, Th=*C*P$_2$), 127.39 (t, $^3J_{PC}$ = 6.0 Hz, C_{meta} of P–Ph), 127.66 (t, $^3J_{PC}$ = 6.0 Hz, C_{meta} of P–Ph), 128.47, 129.39, 129.90, 130.61 (Ar*C*), 132.00 (t, $^3J_{PC}$ = 5.7 Hz, C_{meta} of P–Ph), 132.25 (t, $^3J_{PC}$ = 5.8 Hz, C_{meta} of P–Ph), 137.25 (s, Ar*C*), 137.90 (d, J_{PC} = 42.9 Hz, C_{ipso} of P–Ph), 138.29, 140.92 (Ar*C*), 141.66 (d, J_{PC} = 49.3 Hz, C_{ipso} of P–Ph), 168.80 (s, N*C*Ph$_2$ or ^{t}BuN*C*(N)O), 169.17 (s, N*C*Ph$_2$ or ^{t}BuN*C*(N)O). ^{31}P NMR (C_6D_6, 162 MHz, 298 K): δ (ppm) = 4.37 (s). ^{29}Si{^{1}H} NMR (C_6D_6, 79.5 MHz, 298 K): δ (ppm) = −10.55 (s, N(*Si*Me$_3$)$_2$), −6.03 (t, $^2J_{PSi}$ = 3.50 Hz, P=N(*Si*Me$_3$)). FTIR v/cm^{-1} (Nujol): 1240 (w), 942 (w), 769 (w), 722 (m), 695 (w), 606 (w), 587 (w), 557 (w).

X-ray Crystallography of 1

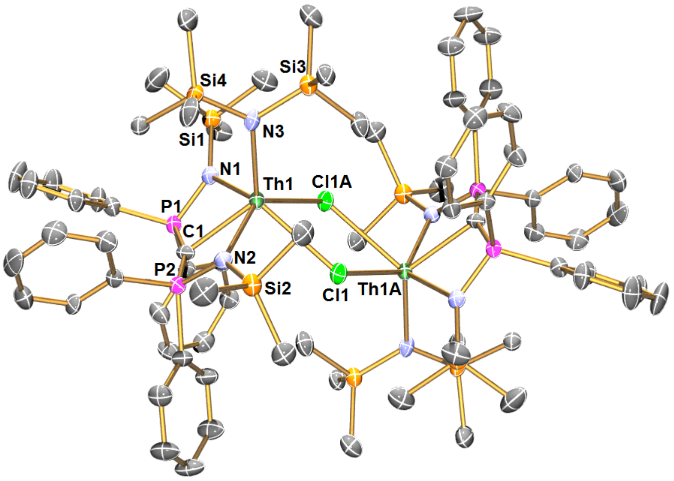

Figure S1. Molecular structures of [Th(BIPM^{TMS}){N(SiMe$_3$)$_2$}(μ-Cl)]$_2$ (**1**). Displacement ellipsoids set at 40% probability. Hydrogen atoms and toluene solvent molecule in lattice are omitted for clarity.

X-ray data for **1-4** have been deposited with the CCDC, numbers 1000534-1000537.

NMR Spectra of 1-4

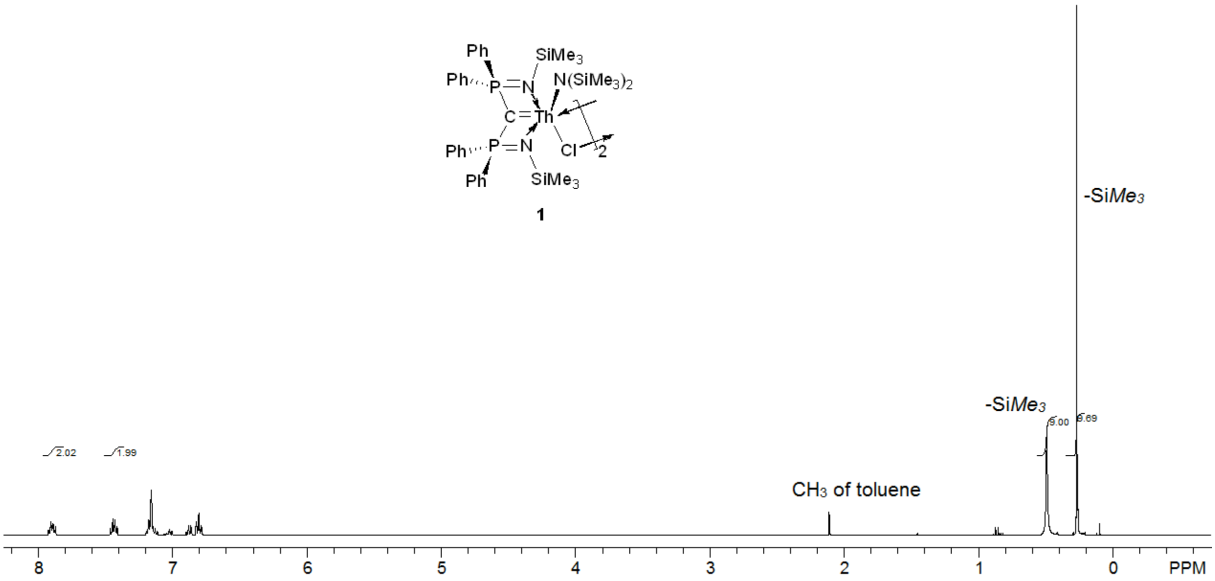

Figure S2. ^{1}H NMR of **1** (C_6D_6, 25 ^oC).

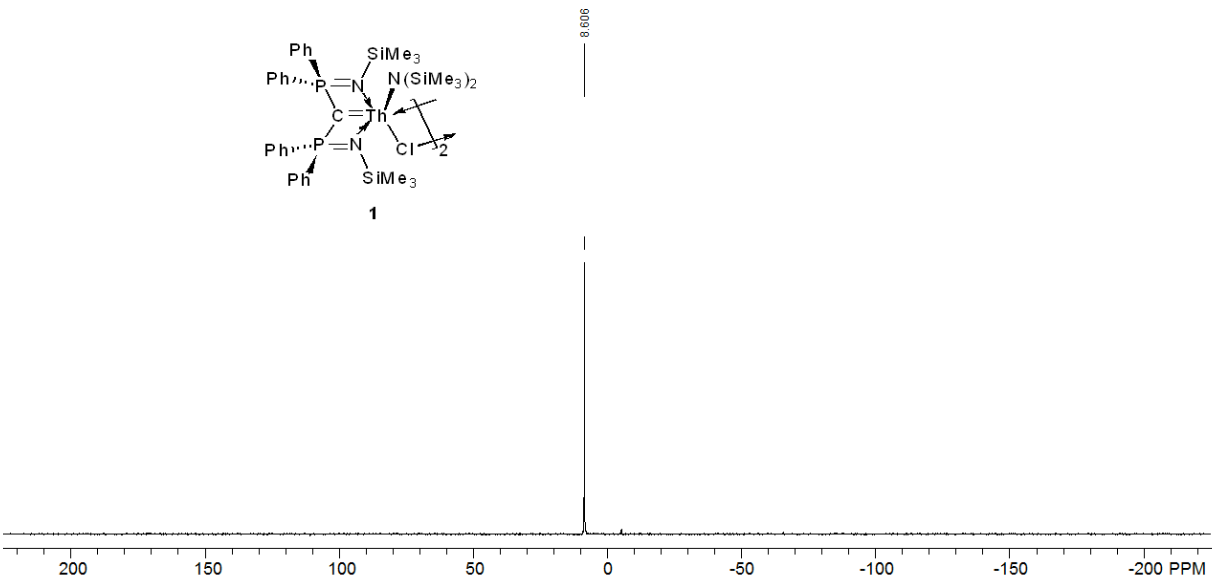

Figure S3 **.** ^{31}P NMR of **1** (C_6D_6, 25 °C).

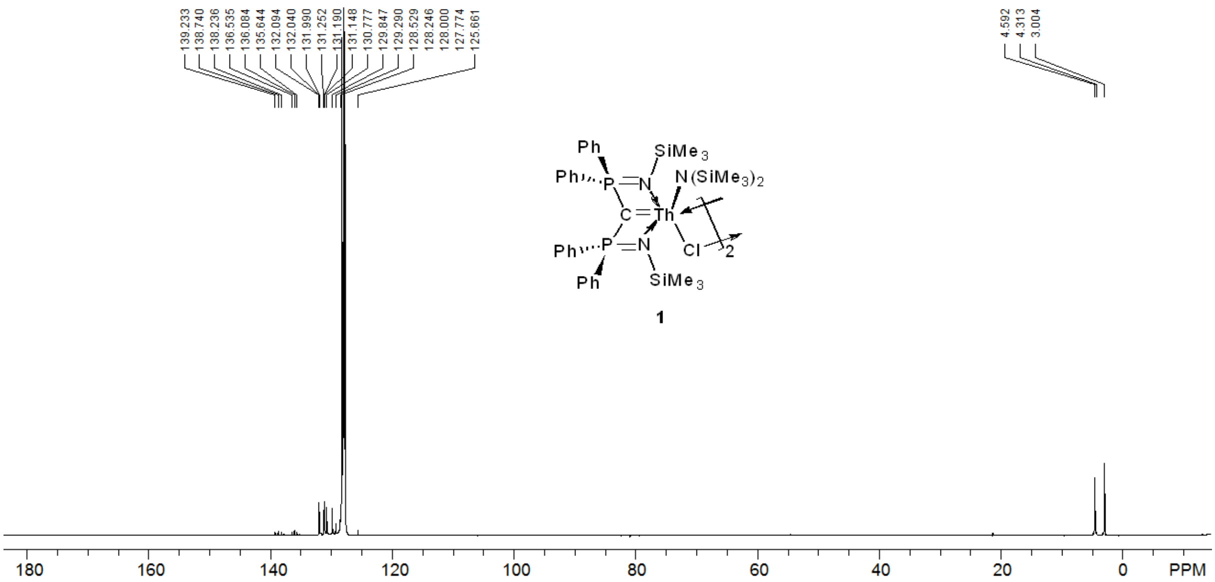

Figure S4. ^{13}C NMR of **1** (C_6D_6, 25 °C).

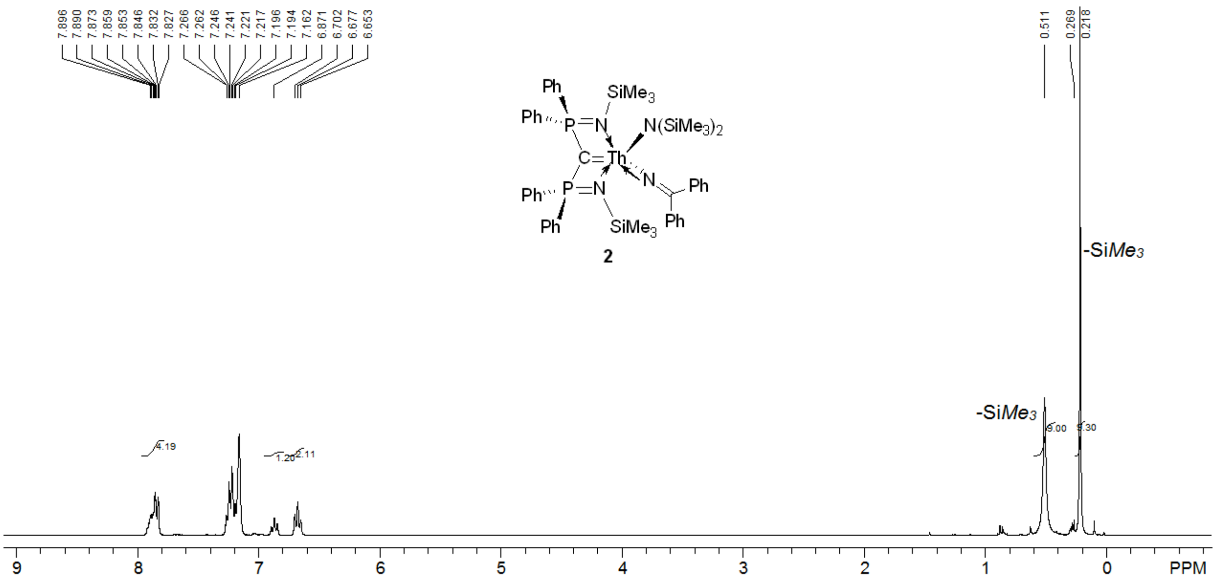

Figure S5. ^{1}H NMR of **2** (C_6D_6, 25 °C).

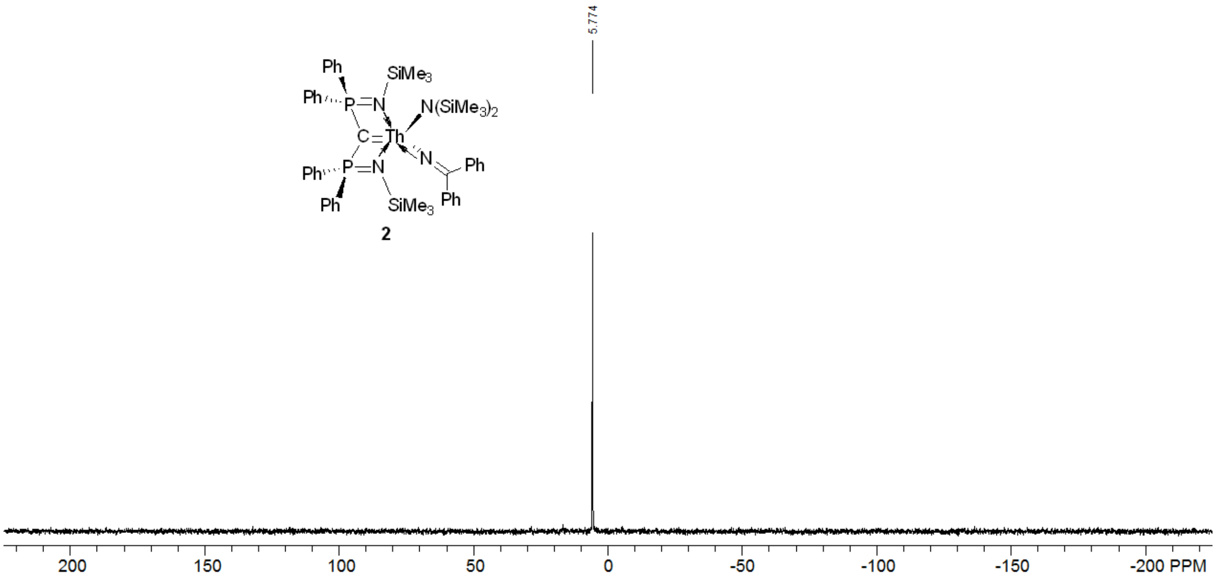

Figure S6. ^{31}P NMR of **2** (C_6D_6, 25 °C).

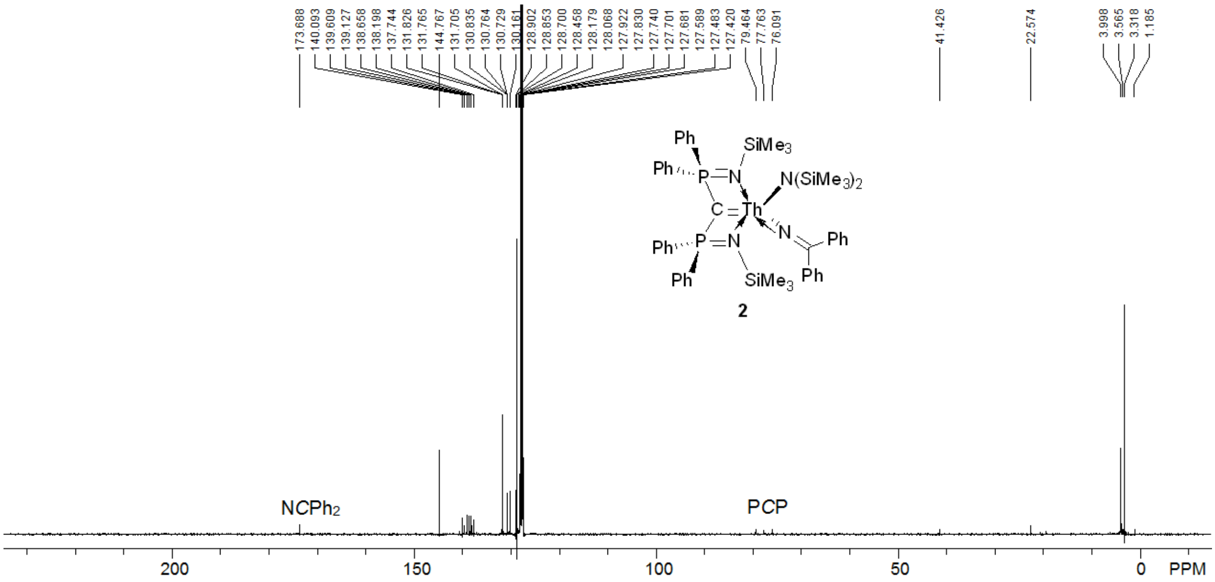

Figure S7. ^{13}C NMR of **2** (C_6D_6, 25 ^{o}C).

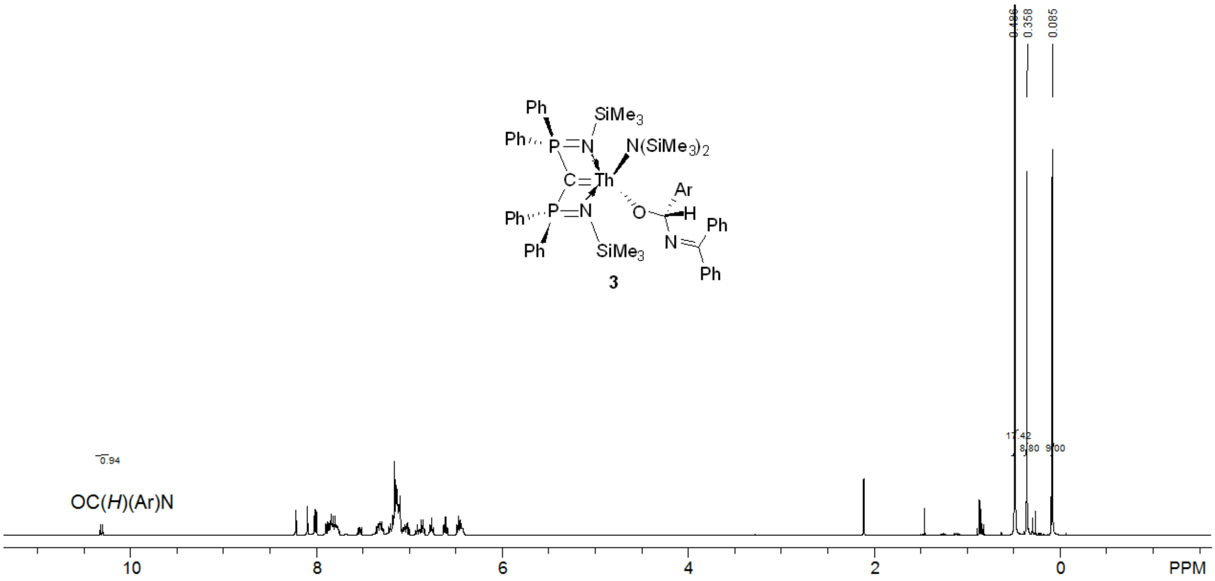

Figure S8. ^{1}H NMR of **3** (C_6D_6, 25 ^{o}C).

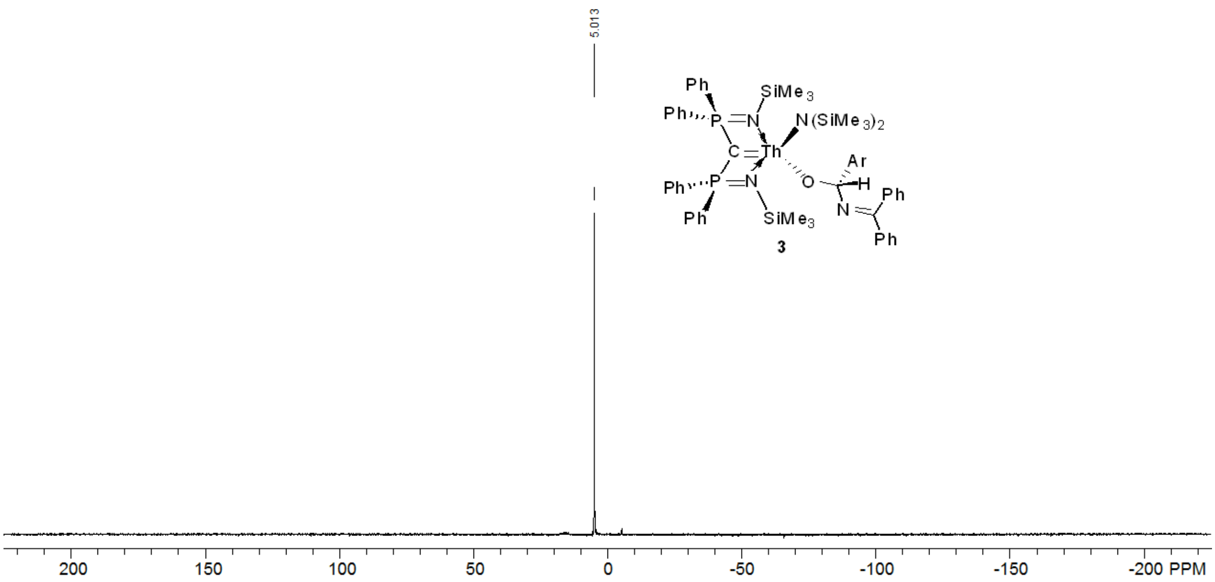

Figure S9. ^{31}P NMR of **3** (C_6D_6, 25 ^{o}C).

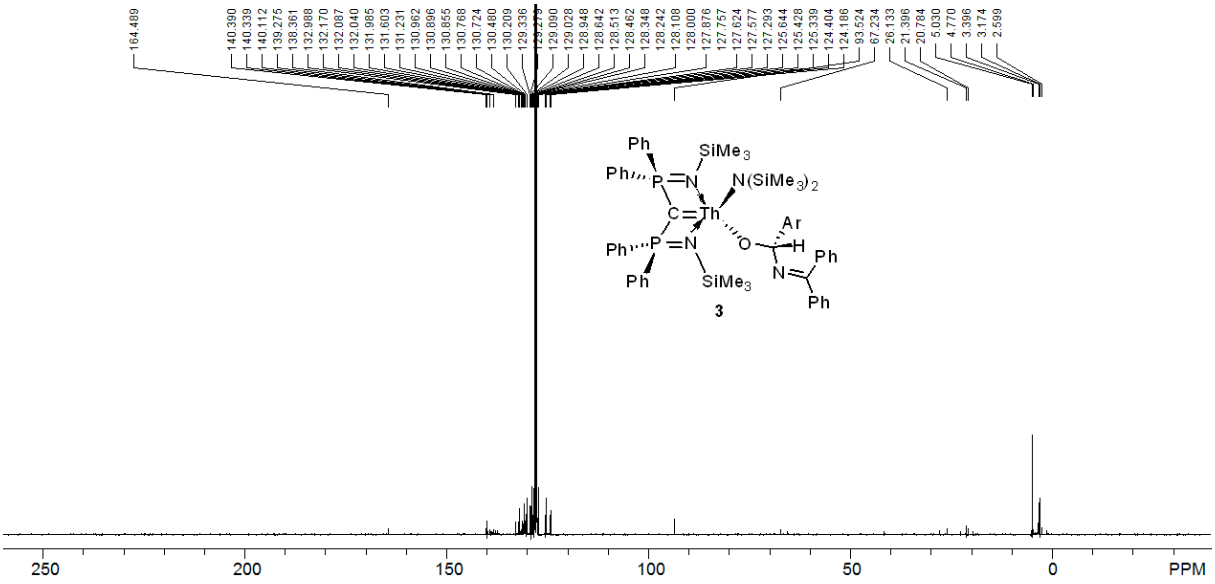

Figure S10. ^{13}C NMR of **3** (C_6D_6, 25 ^{o}C).

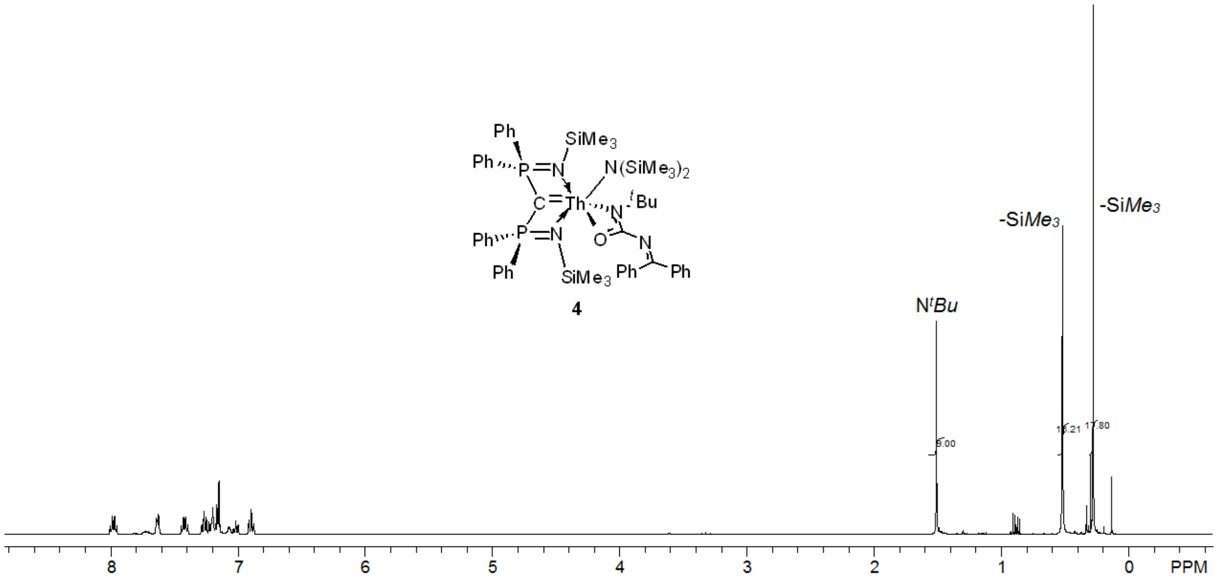

Figure S11. ^{1}H NMR of **4** (C_6D_6, 25 °C).

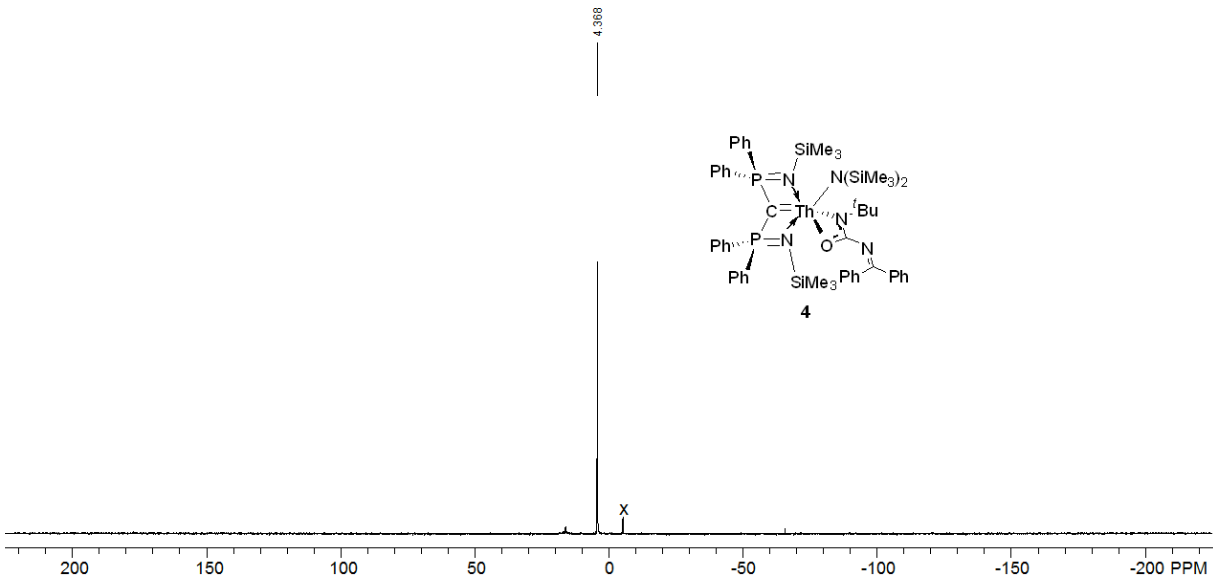

Figure S12. ^{31}P NMR of **4** (C_6D_6, 25 °C). X = small amount of H_2BIPM^{TMS} impurity, which cannot be removed by recrystallization.

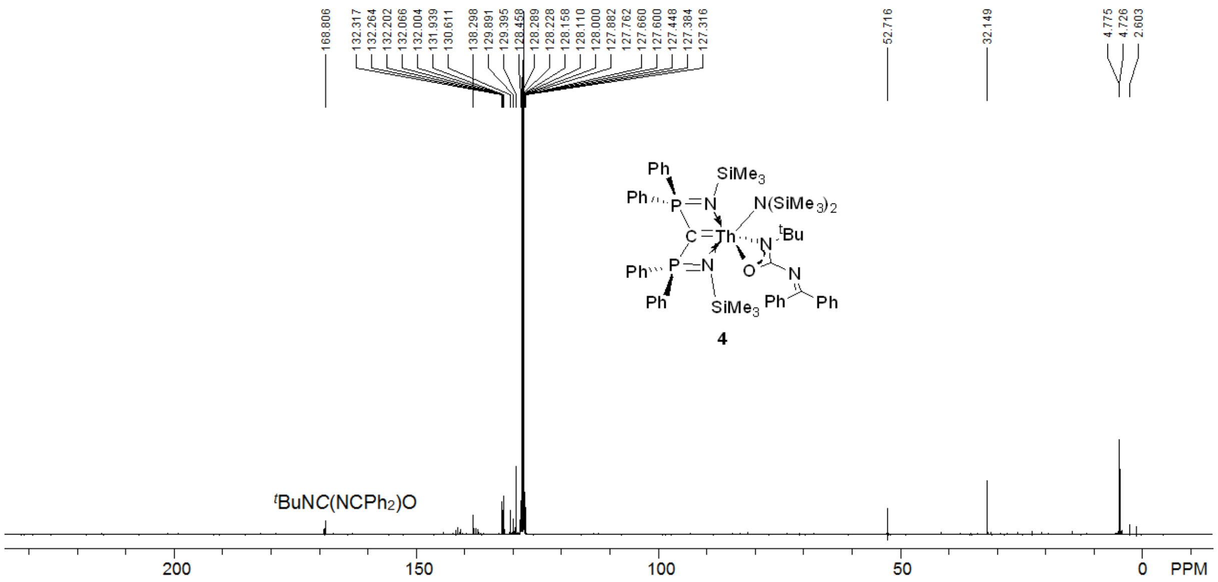

Figure S13. ^{13}C NMR of **4** (C_6D_6, 25 °C).

Electronic Absorption Spectrum of 2

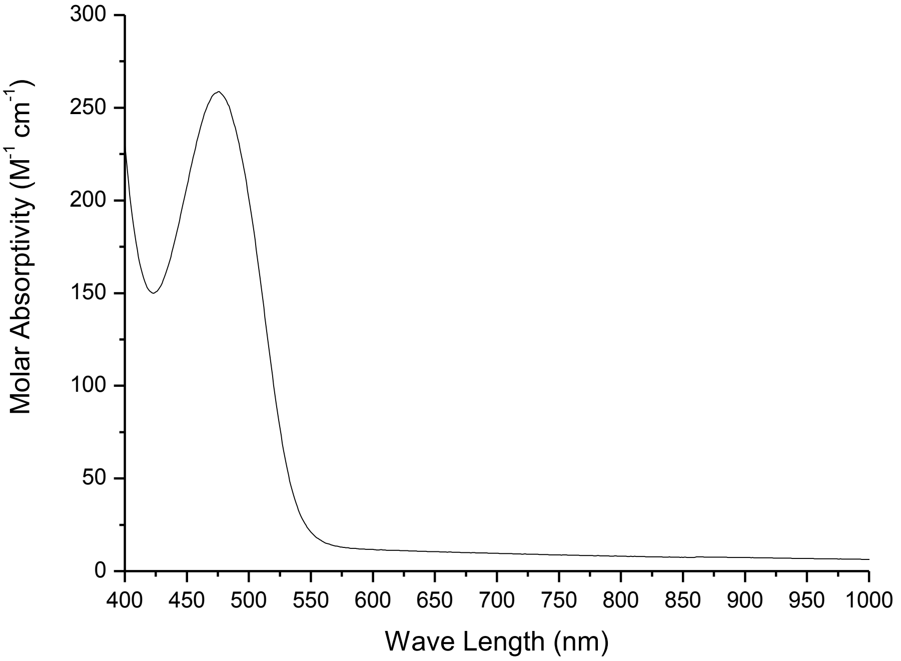

Figure S14. Electronic absorption spectrum of a 10 mM toluene solution of [Th(BIPM^{TMS}){N(SiMe$_3$)$_2$}(N=CPh$_2$)] (**2**).

References

[1] T. Cantat, B. L. Scott, J. L. Kiplinger, *Chem. Commun.* **2010**, *46*, 919.

[2] D. Barr, W. Clegg, R. E. Mulvey, R. Snaith, *J. Chem. Soc., Chem. Commun.* **1984**, 79.
